# Synthesis of *Metarhizium anisopliae*–Chitosan Nanoparticles and Their Pathogenicity against *Plutella xylostella* (Linnaeus)

**DOI:** 10.3390/microorganisms10010001

**Published:** 2021-12-21

**Authors:** Jianhui Wu, Cailian Du, Jieming Zhang, Bo Yang, Andrew G. S. Cuthbertson, Shaukat Ali

**Affiliations:** 1 Key Laboratory of Bio-Pesticide Innovation and Application, South China Agricultural University, Guangzhou 510642, China; jhw@scau.edu.cn (J.W.); ducailian@stu.edu.cn (C.D.); dique@stu.scau.edu.cn (J.Z.); yb@stu.scau.edu.cn (B.Y.); 2 Engineering Research Center of Biological Control, Ministry of Education and Guangdong Province, South China Agricultural University, Guangzhou 510642, China; 3 Independent Science Advisor, York YO10 5AQ, UK; andrew_cuthbertson@live.co.uk

**Keywords:** biopesticides, *Metarhizium anisopliae*, nano-formulation, *Plutella xylostella*

## Abstract

Nanotechnology is increasingly being used in areas of pesticide production and pest management. This study reports the isolation and virulence of a new *Metarhizium anisopliae* isolate SM036, along with the synthesis and characterization of *M. anisopliae*–chitosan nanoparticles followed by studies on the efficacy of nanoparticles against *Plutella xylostella*. The newly identified strain proved pathogenic to *P. xylostella* under laboratory conditions. The characterization of *M. anisopliae*–chitosan nanoparticles through different analytical techniques showed the successful synthesis of nanoparticles. SEM and HRTEM images confirmed the synthesis of spherical-shaped nanoparticles; X-ray diffractogram showed strong peaks between 2θ values of 16–30°; and atomic force microscopy (AFM) analysis revealed a particle size of 75.83 nm for *M. anisopliae*–chitosan nanoparticles, respectively. The bioassay studies demonstrated that different concentrations of *M. anisopliae*–chitosan nanoparticles were highly effective against second instar *P. xylostella* under laboratory and semi-field conditions. These findings suggest that *M. anisopliae*–chitosan nanoparticles can potentially be used in biorational *P. xylostella* management programs.

## 1. Introduction

The diamondback moth (DBM), *Plutella xylostella* (L.) (Lepidoptera: Plutellidae), is one of the most devastating pests of brassicaceous crops resulting in losses of approximately USD 4–5 billion per year worldwide [1]. *Plutella xylostella* is known to rapidly evolve resistance against almost all types of insecticides, including products of *Bacillus thuringiensis* [2]. Consequently, entomopathogenic fungi have received increased attention as potentially an environmentally friendly alternative control measure to insecticides for controlling *P. xylostella*. Several strains of fungi have been isolated and used to control various insect pests, including *P. xylostella* [3,4,5,6,7]. Of these entomopathogenic fungi, *Metarhizium anisopliae* (Cordycipitaceae; Hypocreales) has been documented as an effective pathogen of *P. xylostella* [8,9,10]. Wu et al. [8] studied the pathogenicity of *M. anisopliae* against *P. xylostella*. Their findings showed that three isolates of *M. anisopliae* (M408, M440 and M460) were virulent against *P. xylostella*, having median survival time (ST_50_) values of 1.45, 1.65 and 1.72 days, respectively. In addition to its pathogenicity and ease of mass production, the application of *M. anisopliae* may also have some additional advantages, such as having a broad host range, and safety towards humans and other beneficial organisms [11]. However, various limiting factors such as slower efficacy during the initial few days of field application, the requirement of sophisticated storage conditions and shorter storage time has hindered the large-scale development of *M. anisopliae* as a microbial pesticide [12]. Therefore, the development of highly virulent and environmentally resistant fungal formulations is in high demand.

Nanotechnology is increasingly being used in areas of pesticide production and pest management [13,14]. Nanotechnology can be helpful in enhancing crop yield as well as developing new pest management technologies [15,16]. In recent years, nanocapsules or nanoparticles of crop fortification agents such as pesticides, herbicides, repellents and pheromones have been used as pest control agents [17,18,19,20]. Polymer-based nanomaterials such as chitosan have gained much attention in various areas of agriculture. Chitosan-derived nanoparticles have possible biological activities due to their biodegradable and nontoxic properties [14]. Chitosan nanoparticles are generally formulated by physical and chemical methods and are globally used for numerous applications including agriculture and drug delivery. The effective biocontrol potential of chitosan-derived nanoparticles of different microbial control agents have been suggested for their formulation and application as natural pest control agents [21]. The effective biocontrol potential of chitosan nanoparticles against plant pathogens such as *Fusarium solani* associated with various economic important crops suggested the formulation and application of chitosan nanoparticles as a natural antifungal agent to enhance its antifungal activity [22]. Namasivayam et al. [14] observed enhanced pesticidal activity and biocompatibility of chitosan nanocomposite prepared with the chitosan–fungal biopesticidal agent *Nomuraea rileyi* against *Spodoptera litura.* This study focuses on the synthesis, characterization and toxicity of *M. anisopliae*–chitosan nanoparticles, which will enhance knowledge on the development of nanoformulations of entompathogenic fungi by mixing their conidia with other nanomaterials such as chitosan. These properties will allow decreased pesticide doses and achieve greater pest control without repeated treatments [23]. Some circles of the scientific community still think that the use of nanopesticides is not fully safe as chemical entities with unknown physicochemical and toxicological properties are released into the environment [24]. However, the use of quantitative structure–activity relationship/quantitative structure–property relationship (QSAR/QSPR) tools offer viable solutions to address experimental issues observed during pesticide risk assessment [25].

The main objectives of this study were: (a) To identify *M. anisopliae* isolate SM036; (b) Synthesis and characterization of *M. anisopliae*–chitosan nanoparticles through different analytical techniques; and (c) To observe the toxicity of the developed *M. anisopliae*–chitosan nanoparticles (liquid suspension) against second instar *P. xylostella* larvae under laboratory and semi-field conditions.

## 2. Materials and Methods

### 2.1. Insect Rearing

Adults of *P. xylostella* were obtained from stock cultures reared on *Brassica oleracea* (Brassicaceae) following Ali et al. [4] and kept in a greenhouse at the Engineering Research Center of Biological Control, South China Agricultural University, Guangzhou, Guangdong, China. Plants were grown in plastic pots with a diameter of 15 cm. Sufficient slow-release fertilizer (N:P:K = 13:7:15; Shenzhen Batian Ecotypic Engineering, Shenzhen, China) was added as required to maintain normal plant growth. The newly molted second instars of *P. xylostella* were gently removed from the host plant leaves using a fine camel-hair brush (No. 00) and placed on pieces of fresh *B. campestris* leaf (100–150 cm^2^).

### 2.2. Collection, Isolation and Identification of Metarhizium anisopliae

Samples of sandy loam soil were collected from cotton fields of Qapqal Xibe autonomous county, Xinjiang Autonomous Region, P.R. China in 2008. The samples were collected to a depth of 10 cm under the surface soil layer and placed into sealed polyethylene bags after excavation. Isolation of fungal samples was carried out according to the methods of Imoulan et al. [26] and Du et al. [27]. Any spore-like growth was inoculated onto new medium for the purification of fungal cultures. The culture purification was performed until the growth of a single colony on potato dextrose agar (PDA) plates.

The genomic DNA of the isolated fungal strain SM036 was extracted using a fungal DNA isolation kit purchased from Ezup, Sangon Biotech, (Shanghai, China). ITS fragments were amplified by PCR using the genomic DNA as a template by using ITS4F (TCCTCCGCTTATTGATATGC) and ITS5R (GGAAGTAAAAGTCGTAACAAGG) primers. The PCR cycling and conditions as outlined in Rehner et al. [28] were used for amplification of the ITS genes. The amplified PCR amplification products were detected by agarose gel electrophoresis. The amplified products with correct size were sequenced by Tsingke Biotechnology Co., Ltd. (Beijing, China).

The sequencing results were spliced by Shengxin software Geneious 7.1.4 and the homology of genes was compared by the NCBI online program blast from which the corresponding sequences of published model strains were downloaded. The phylogenetic tree was constructed by using the Evolutionary Analysis Software Mega 7.0. Neighbor-joining method was used to construct trees, and the bootstrap method was used to select parameters of 1000. Finally, the substitution model was used to select p-distance.

### 2.3. Pathogenicity of Metarhizium anisopliae Isolate SM036 against Plutella xylostella

The conidial suspensions of *M. anisopliae* SM036 with different concentration gradients (1 × 10^8^, 5 × 10^7^, 1 × 10^7^, 5 × 10^6^, 1 × 10^6^, 5 × 10^5^ and 1 × 10^5^ conidia/mL) were prepared following the method of Ali et al. [4]. Briefly, conidia were harvested from PDA plates with deionized water containing 0.03% Tween-80 and sieved using filter paper (Whatman no. 2; Science Kit and Boreal Laboratories, New York, NY, USA) into sterile vials. Conidia were counted using a compound microscope (Axio Lab. A1, Zeiss, Germany) and a hemocytometer (0.0625 m^2^; Fuchs-Rosenthal Merck Euro Lab, Darmstadt, Germany) to calibrate a suspension of 1 × 10^8^ conidia/mL. Lower concentrations were prepared by serial dilution using deionized water containing 0.03% Tween-80 as solvent.

Newly hatched 2nd instar *P. xylostella* larvae (30 individuals) were moved to cabbage leaves (obtained from a 60-day-old plant) via a camel hairbrush. The 2nd instar *P. xylostella* larvae were subjected to the above varying concentrations of *M. anisopliae* SM036. An application of ddH_2_O served as an untreated control. The fungal suspensions were sprayed on leaves for 30 s to run-off using an automizer sprayer (Nongbao 4174191 sprayer, Anhui, China) [18]. The leaves were allowed to air dry before being placed into a Petri dish (ø 9 cm) having moist filter paper for moisture maintenance. The whole experimental setup was maintained at 25 ± 1 °C and 80 ± 5% R.H and 16:8 h (Light:Dark). The experiment was performed three times (using a fresh batch of insects each time). Insect mortality was recorded on a daily basis for 7 days post application. To confirm mortality caused by *M. anisopliae*, the cadavers were taken out and separately placed at 26 °C and >95% R.H in a growth chamber (RXZ-500D, NingBo JiangNan Equipments, Ningbo, China). If conidia and conidiophore of *M. anisopliae* were observed from a cadaver, the cadaver was regarded as dead from infection of *M. anisopliae*. The conidia and conidiophore of *M. anisopliae* were observed under a phase contrast microscope (Axio Lab. A1, Zeiss, Jena, Germany) at 40× magnification.

### 2.4. Synthesis of Metarhizium anisopliae–Chitosan Nanoparticles

*Metarhizium anisopliae* conidial powder (2 g) was added to 20 mL chitosan solution (0.35% *w*/*v* prepared by dissolving chitosan powder in 1.5% acetic acid) followed by drop-wise addition of 12 mL sodium tripolyphosphate (0.4%). The whole mixture was then stirred for 6 h at room temperature on a magnetic stirrer (Rexim RP-1DN, Agile). The obtained homogenous slurry was added to a screw cap vial and incubated at 45 °C for 12 h. The change in color of solution from colorless to yellowish brown confirmed nanoparticle synthesis. The synthesized reaction mixture was centrifuged for 10 min at 15,000× *g* and 28 °C (Minispinplus centrifuge, Eppendorf, Hamburg, Germany). Pellets obtained were washed with deionized water followed by freeze drying in a refrigerator at −20 °C (BCD-576, Haier, Qingdao, China) until further use.

### 2.5. Characterization of Metarhizium anisopliae–Chitosan Nanoparticles

The viability of *M. anisopliae*–chitosan nanoparticles was observed by the inoculation of 0.2 mL of 125 ppm nanoparticles solution or 0.2 mL of 1 × 104 conidia/mL of *M. anisopliae* on PDA plates. The number of germinated propagules was counted every 24 h. Propagules were considered viable when the germ tube lengths corresponded to width. The extracellularly synthesized *M. anisopliae*–chitosan nanoparticles were characterized through various analytical techniques used for nanoparticle characterization, as outlined in previous studies [18,20,29,30]. To perform scanning electron microscopy, samples were processed and fixed by following the method of Wang et al. (2019). The images were captured under SU8010 (Hitachi, Japan) SEM operating at an accelerated voltage of 5.0 kV. The samples for high-resolution transmission electron microscopy (HRTEM) were prepared by applying a drop of the aqueous suspension of films on carbon-coated copper grids. The sample, grounded into small pieces, was added to liquid nitrogen so that the depth of the resolution could be improved. A JEOL JEM 1200 EXII microscope attached with a UHR pole piece and a lattice resolution of 0.14 nm with the accelerating voltage of 120.0 kV was used to determine the image of the nanomaterials.

The nanoparticles were characterized qualitatively through FTIR analysis using a MIR8035 FTIR spectrometer (Thermo Fisher, Bremen, Germany). X-ray diffractometry analysis was undertaken (using Cu-Kα radiations in a Bruker D8 diffractometer, Germany) for the calculation of the crystalline structure of *M. anisopliae*–chitosan nanoparticles. Atomic force microscopy (AFM) analysis was undertaken to study the surface topology. On a glass slide, 100 μL of the respective sample was placed in order to obtain a thin film of the sample. The sample was then allowed to dry for 5 min. AFM Dimension Icon-Bruker, Germany was instrumental in scanning the slides. Zeta potential (surface charge) of *M. anisopliae*–chitosan nanoparticles was analyzed by using the Zetasizer (Malvern, UK) at 25 °C.

### 2.6. Biological Activity of Chitosan-Based Nanoparticles of Metarhizium anisopliae against Plutella xylostella under Laboratory Conditions

The biological activities of chitosan-based *M. anisopliae* nanoparticles were tested against 2nd instar *P. xylostella* larvae under laboratory conditions by following Xu et al. [19]. Newly hatched 2nd instar *P. xylostella* larvae (30 individuals) were moved to cabbage leaves (obtained from a 60 day old plant) via a camel hairbrush. The 2nd instar *P. xylostella* larvae were subjected to five different concentrations of *M. anisopliae*–chitosan nanoparticles (named as T1-T5 in Table 1). The 2nd instar *P. xylostella* larvae were also treated with a conidial suspension (1 × 10^6^ conidia/mL) of *M. anisopliae* (T6) to compare the bioactivity of *M. anisopliae*–chitosan nanoparticles with fungal conidia (can also be termed as positive control) whereas application of 200 ppm chitosan (T7) and ddH_2_O (T8) served as controls. The suspensions were sprayed on leaves for 30 s to run-off using an automizer sprayer [18]. The leaves were allowed to air dry before being placed into a Petri dish (ø 9 cm), having moist filter paper for moisture maintenance. The whole experimental setup was maintained at 25 ± 1 °C and 80 ± 5% R.H and 16:8 h (Light:Dark). The experiment was performed three times (using a fresh batch of insects each time). Insect mortality was recorded on a daily basis for 7 days post application.

### 2.7. Biological Activity of Chitosan-Based Nanoparticles of Metarhizium anisopliae against Plutella xylostella under Field Conditions

Newly molted 2nd instar larvae were gently moved to the leaves of cabbage plants (60 days old), with 3 larvae for one leaf per plant. The 2nd instar *P. xylostella* larvae were subjected to five different concentrations of *M. anisopliae*–chitosan nanoparticles (named as T1-T5 in Table 1). The 2nd instar *P. xylostella* larvae were also treated with a conidial suspension (1 × 10^6^ conidia/mL) of *M. anisopliae* (T6) to compare the bioactivity of *M. anisopliae*–chitosan nanoparticles with fungal conidia (can also be termed as positive control) whereas application of 200 ppm chitosan (T7) and ddH_2_O (T8) served as controls. The different test suspensions were sprayed as described in Section 2.6. Each plant with a treated leaf was covered with a plastic screen in the field. Each treatment had 30 larvae, and the entire experiment was conducted three times on different dates. Each application was commenced at 1700 h. Insect mortality was recorded on a daily basis for 7 days post application.

### 2.8. Data Analysis

Insect mortality values were percentage transformed followed by probit analysis [31]. SAS 9.2 software was used for all data analysis [32].

## 3. Results

### 3.1. Isolation and Identification of Metarhizium anisopliae Isolate SM036

*Metarhizium anisopliae* isolate SM036 was successfully isolated from the collected soil samples. Its morphological characteristics and sporulation structure on PDA plates were successfully observed. The colony surface of the *M. anisopliae* isolate SM036 was villous, initially white and olive green during sporulation. The mature conidia were agglomerated into shells and were spread on the surface of colonies. The morphology of conidia was long columnar, about 3.5–4.6 μm × 2–3.1 μm, in a chain shape. The length of mycelium was 21–23 μm (Supplementary Figure S1).

**Figure 1:**
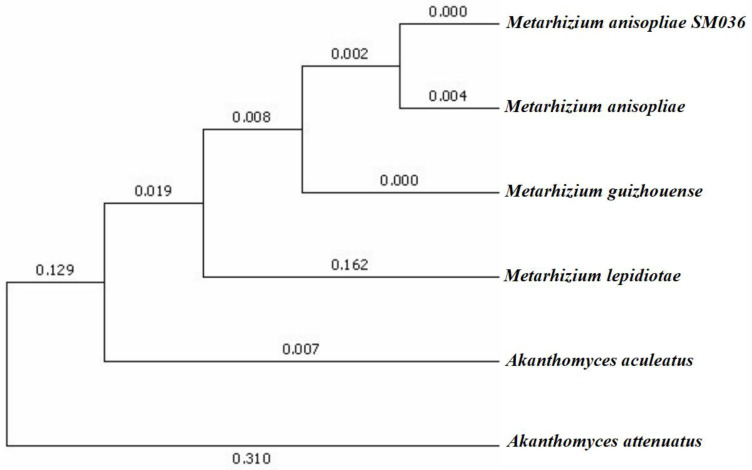
Neighbor-joining (NJ) tree of *Metarhizium anisopliae* SM036 isolate.

An ITS fragment was produced by DNA amplification of fungus strain SM036 and the length of the ITS sequence was 565bp, 1011bp, 956bp, 828bp, respectively. Multiple comparisons were performed by Clustal W and phylogenetic trees were constructed separately according to the Neighbor-joining method. *Akanthomyces* was used as an outgroup. The ITS sequence of *M. anisopliae* SM036 (Genbank# MZ150506) had 100% similarity and *E-value* of 0.00 with *M. anisopliae* (Genbank# MN078271) (Figure 1).

### 3.2. Concentration–Mortality Response of Plutella xylostella to Metarhizium anisopliae Isolate SM036

**Figure 2:**
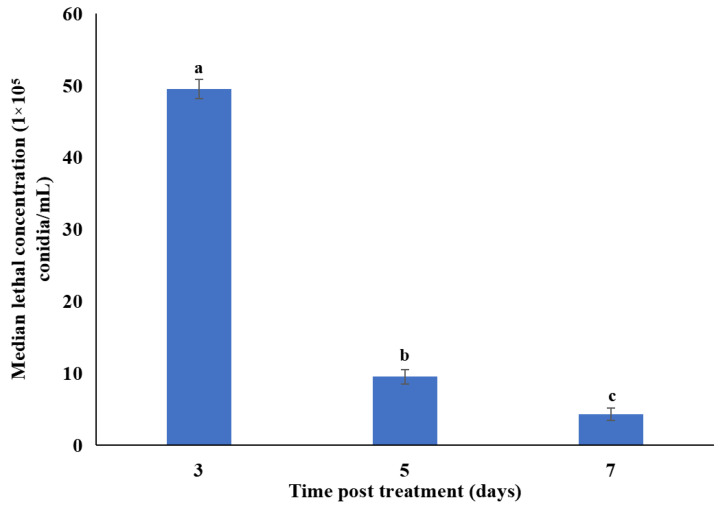
Median lethal concentrations (LC_50_) of *Metarhizium anisopliae* SM036 against second instar *Plutella xylostella* larvae.Error bars indicate the standard error of the means based on three replicates. Bars with distinct letters at different days post treatment differed significantly from each other.

The cumulative daily mortality of second instar *P. xylostella* larvae treated with a conidial suspension of *M. anisopliae* SM036 in response to different concentrations is shown in Supplementary Figure S2. The mortalities of second instar *P. xylostella* larvae were significantly different at 3 (F_7,16_ = 24.17; *p* < 0.001), 5 (F_7,16_ = 39.71; *p* < 0.001), and 7 days (F_7,16_ = 32.65; *p* < 0.001) post treatment with different concentrations of *M. anisopliae.* No significant differences were noted between mean adjusted mortality observed for conidial concentrations of 5 × 10^7^ and 1 × 10^8^ conidia/mL after 3, 5 and 7 days of treatment. Control DBM mortalities were 2.33%, 3.67% and 5.43%, respectively, at 3, 5 and 7 days post-treatment. The median lethal concentrations (LC_50_) of *M. anisopliae* against second instar *P. xylostella* larvae after 7 days of treatment was 4.3 × 10^5^ conidia/mL (Figure 2).

### 3.3. Characterization of Metarhizium anisopliae–Chitosan Nanoparticles

**Figure 3:**
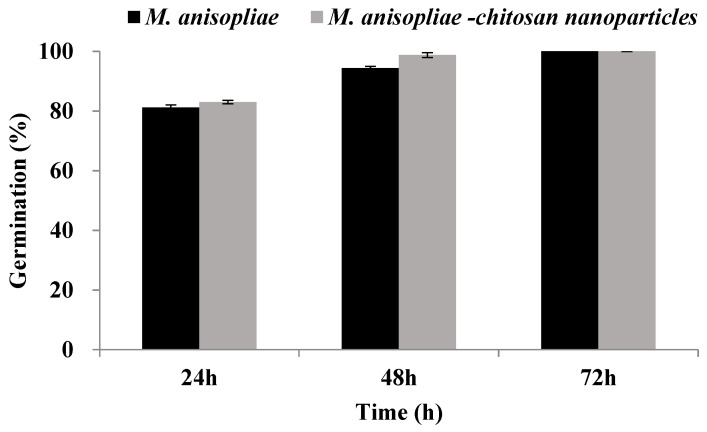
Germination percentage of *Metarhizium anisopliae*–chitosan nanoparticles and *M. anisopliae* conidia at different time periods. Error bars indicate the standard error of the means based on three replicates.

Assays of fungal viability carried out in PDA medium showed that germination rates (%) of *M. anisopliae*–chitosan nanoparticles after 24, 48 and 72 h of inoculation were 83.01, 98.76 and 100%, respectively. In comparison, the germination rate of *M. anisopliae* conidia was 81.20, 94.40 and 100% after 24, 48, and 72 h of growth (Figure 3).

**Figure 4:**
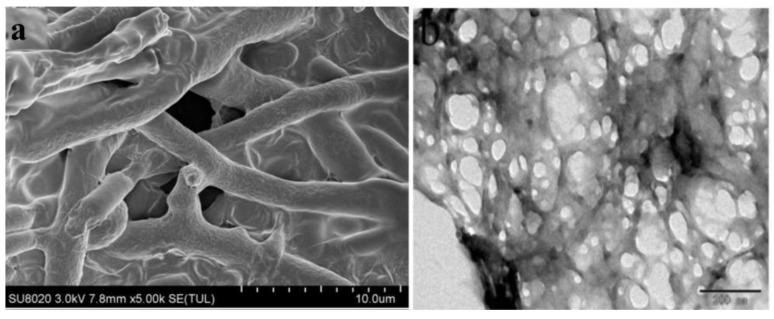
Scanning electron microscopy (**a**) and high-resolution transmission electron microscopy (**b**) of *Metarhizium anisopliae*–chitosan nanoparticles.

Field emission scanning electron microscopy (FESEM) can clearly show the shapes of free chitosan nanoparticles and *M. anisopliae*–chitosan nanoparticles. The size of *M. anisopliae*–chitosan nanoparticles was 10–28 µm (Figure 4a). High-resolution transmission electron microscopy (HRTEM) of the nanocomposites clearly revealed the structural changes and specific interactions between the fungus and chitosan. As chitosan nanoparticles were coated with *M. anisopliae*, the internal structure of nanoparticles was granular (Figure 4b).

**Figure 5:**
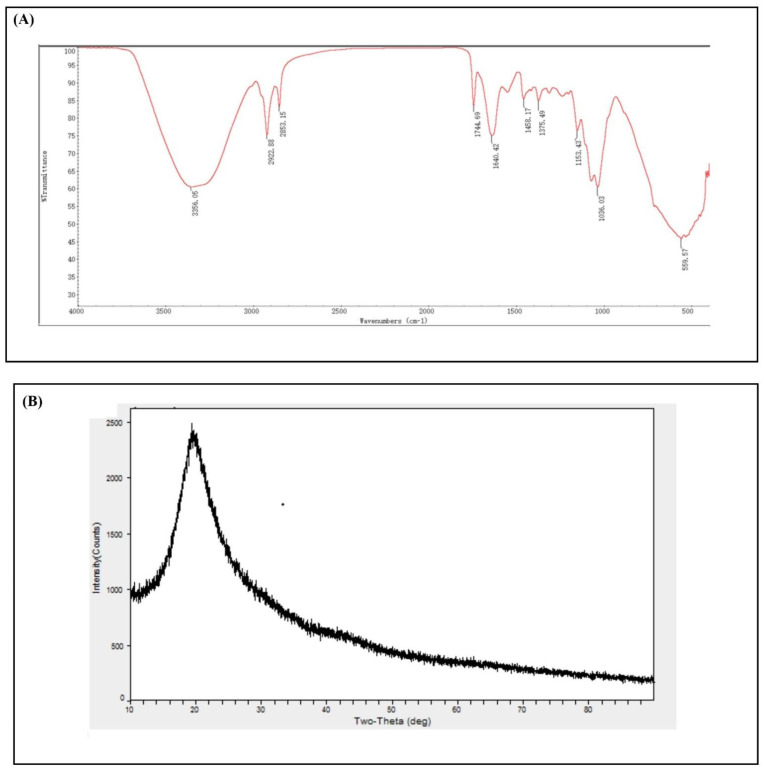
Fourier transform infrared spectroscopy (**A**) and X-ray diffraction crystallography (**B**) of *Metarhizium anisopliae*–chitosan nanoparticles.

The main characteristics of the synthesized *M. anisopliae*–chitosan nanoparticles were analyzed by FTIR (Figure 5a). In the FTIR profiles of *M. anisopliae*–chitosan nanoparticles, O-H or N-H stretching was observed at 3356.50 cm^−1^. The amide linkage peaks for *M. anisopliae*–chitosan nanoparticles were observed at 1640.20 cm^−1^.

The X-ray diffraction patterns of *M. anisopliae*–chitosan nanoparticles showed a strong specific peak at 2θ = 16–30°; this is a unique aspect of the amorphous structure (Figure 5b).

**Figure 6:**
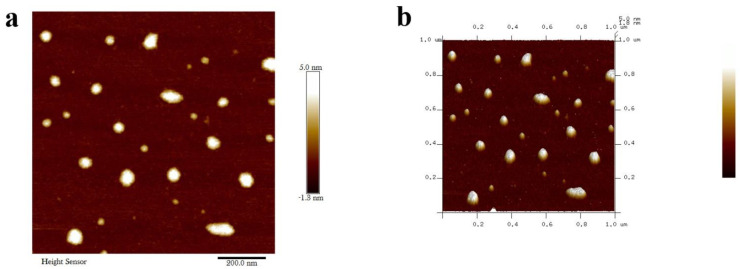
Two-dimensional atomic force microscopy (**a**) and three-dimensional atomic force microscopy (**b**) of *Metarhizium anisopliae*–chitosan nanoparticles.

Atomic force microscopy (AFM) images showed the surface topological structure of chitosan nanocomposites at two-dimensional and three-dimensional levels. The average size of *M. anisopliae*–chitosan nanoparticles was 75.83 nm (Figure 6).

**Figure 7:**
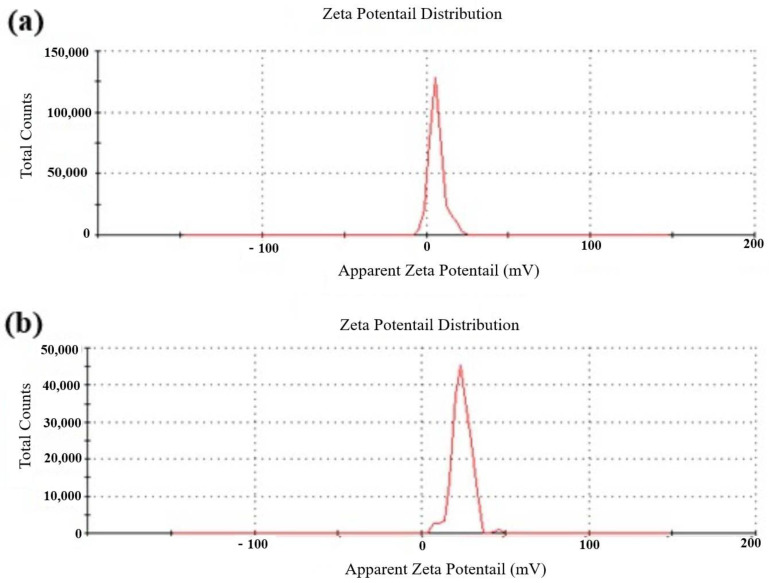
Particle size analysis of *Metarhizium anisopliae* conidia (**a**), and *M. anisopliae*–chitosan nanoparticles (**b**).

The zeta potential of *M. anisopliae* conidia was 6.22 mV (Figure 7a), whereas the zeta potential of *M. anisopliae*–chitosan nanoparticles was 23.27 mV (Figure 7b).

### 3.4. Biological Activity of Chitosan-Based Nanoparticles of Metarhizium anisopliae against Plutella xylostella under Laboratory Conditions

**Figure 8:**
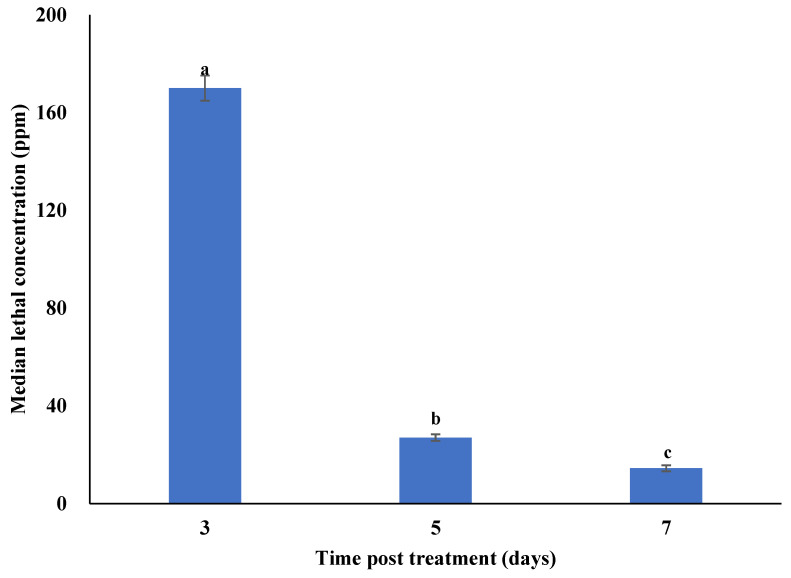
Median lethal concentrations (LC_50_) of *Metarhizium anisopliae*–chitosan nanoparticles against *P. xylostella* under laboratory conditions. Error bars indicate the standard error of the means based on three replicates. Bars with distinct letters at different days post treatment differed significantly from each other.

The mean cumulative daily mortality of second instar *P. xylostella* larvae was directly proportional to the concentration and time post treatment (Supplementary Figure S3). The mortality of second instar *P. xylostella* larvae under laboratory conditions was significantly different at 3 (F_7,16_ = 45.72; *p* < 0.001), 5 (F_7,16_ = 39.34; *p* < 0.001), and 7 (F_7,16_ = 30.16; *p* < 0.001) days post treatment. The highest rates of cumulative mortality in response to different concentrations of *M. anisopliae*–chitosan nanoparticles at 3, 5 and 7 days post treatment were observed for the 500 ppm concentration. No significant differences were observed between cumulative mortality observed for 125, 62.5, and 31.25 ppm at 3 days post treatment. The median lethal concentrations (LC_50_) of *M. anisopliae*–chitosan nanoparticles against second instar *P. xylostella* larvae after 3, 5 and 7 days of treatment were 170.01, 26.99, and 14.44 ppm, respectively (Figure 8).

### 3.5. Biological Activity of Chitosan-Based Nanoparticles of Metarhizium anisopliae against Plutella xylostella under Field Conditions

**Figure 9:**
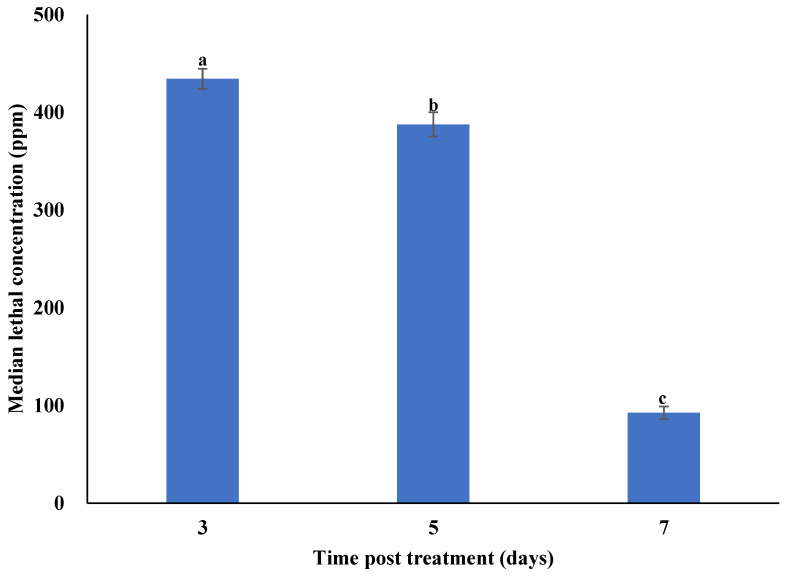
Median lethal concentrations (LC_50_) of *Metarhizium anisopliae*–chitosan nanoparticles against *P. xylostella* under field conditions. Error bars indicate the standard error of the means based on three replicates. Bars with distinct letters at different days post treatment differed significantly from each other.

The mean cumulative daily mortality of second instar *P. xylostella* larvae was directly proportional to the concentration and time post treatment (Supplementary Figure S4). The mortality of second instar *P. xylostella* larvae under field conditions was significantly different at 3 (F_7,16_ = 29.06; *p* < 0.001), 5 (F_7,16_ = 31.42; *p* < 0.001), and 7 (F_7,16_ = 38.26; *p* < 0.001) days post treatment. The highest rates of cumulative mortality in response to different concentrations of *M. anisopliae*–chitosan nanoparticles at 3, 5 and 7 days post treatment were observed for the 500 ppm concentration. The median lethal concentrations (LC_50_) of *M. anisopliae*–chitosan nanoparticles against second instar *P. xylostella* larvae after 3, 5, and 7 days of treatment were 434.42, 387.73, and 92.69 ppm, respectively (Figure 9).

## 4. Discussion

### 4.1. Isolation and Identification of Metarhizium anisopliae Isolate SM036

A proper classification/identification of insect pathogenic microorganisms is a basic/initial requirement for their development as biopesticides [24]. The identification of fungi is mainly carried out through the comparison of the internal transcribed spacer fragments (ITS) of DNA [33]. The maximum likelihood tree constructed based on ITS sequences classified the new isolate as *Metarhizium anisopliae*. The mycelial color of the *M. anisopliae* isolate observed here was consistent with the characteristic *M. anisopliae* isolates QU-M421, QUM171a, QU-M363, and Qu-M430 observed by Sepulveda et al. [34]. However, the length × width of conidia recorded here were smaller than the length × width of *M. anisopliae* conidia described by Bischoff et al. [35] and Fernandes et al. [36]. The colony diameter (32.00 mm) and conidia yield (4.7 × 10^7^ conidia/mL) observed during this study were similar to Wu et al. [8] who observed a colony diameter of 32.00 mm and conidial yield of 4.01 × 10^7^ conidia/mL for *M. anisopliae* isolate M440 when grown on PDA medium.

### 4.2. Concentration–Mortality Response of Plutella xylostella to Metarhizium anisopliae Isolate SM036

The present study highlights the virulence of *M. anisopliae* against second larval instar of *P. xylostella*. *Metarhizium anisopliae* displayed lethal effects against *P. xylostella,* as was reported in previous studies conducted by Loc and Chi [37] and Zafar et al. [10]. The results depict that the virulence was strongly related to concentration exposed to immatures, and post-treatment time. Overall, an increase in fungal concentration was found to be more potent against *P. xylostella*. The results obtained by Mehinto et al. [38] are in accordance with our findings, where 1 × 10^8^ spores/mL showed peak mortality against *Maruca vitrata* (Lepidoptera: Crambidae). In addition, Freed et al. [39] also reported that high EPF concentrations against DBM prompted significantly higher mortality as compared to low concentrations. Xia et al. [40] found the same virulence pattern in *P. xylostella*. However, non-significant mortality was observed in the case of low concentrations. The virulence of the fungal strain depends on both the number and growth of spores within the host body [41]. The sporulation has a direct relationship with host mortality, as suggested by Pauli et al. [42].

### 4.3. Characterization of Chitosan-Based Nanoparticles of Metarhizium anisopliae

The utilization of nano-materials in conjunction with microbial pest control agents can add a new dimension to insect pest control [20,21,22]. Nanoparticles of entomopathogenic fungi synthesized by the addition of different metals (Ag, Cu, Au or Fe) or other nano-materials (Biochar, chitosan) have shown considerable toxicity against different insect pests [19,20,21,43]. During our previous studies, *Beauveria brongniartii*–Fe^0^ nanoparticles (500 ppm) caused 100% mortality of second instar *Spodoptera litura* larvae, as well as a significant reduction in the feeding and growth parameters of *S. litura* [19]. This study reports the production and characterization of *M. anisopliae*–chitosan nanoparticles. The FTIR profiles of synthesized chitosan nanoparticles, *M. anisopliae* toxin–chitosan nanoparticles and *M. anisopliae* hyphae–chitosan nanoparticles showed the presence of O-H or N-H stretching and carbonyl stretch amide linkage, which is similar to the FTIR profile of *Noumeraea rileyi*–chitosan nanoparticles, explained by Namasivayam et al. [14]. The changes in O-H or N-H stretching and carbonyl stretch amide linkage of nanoparticles in this study confirmed structural changes in response to the chitosan coating of metabolites or fungal conidia [44]. The XRD patterns of chitosan nanocomposites prepared by chitosan nanoparticles, metabolites or mycelium had a strong specific peak at 2θ = 16–30° corresponding to the amorphous structure calculated through Bragg’s law [45] and Miller indices [46]. The XRD peak observed in this study was consistent with the findings of Namasivayam et al. [14], who observed similar XRD profiles for *N. rileyi* chitosan nanoparticles. The atomic force microscopy (AFM) was used to study the surface topology of prepared nanoparticles. The average size of *M. anisopliae* toxin–chitosan nanoparticles and *M. anisopliae* mycelium–chitosan nanoparticles observed during this study was smaller than the average size of *N. rileyi*–chitosan nanoparticles (85.1) observed by Namasivayam et al. [14]. Zeta potential is an important parameter affecting its suspension stability. The zeta potential values observed for *M. anisopliae*–chitosan nanoparticles were different from Namasivayam et al. [14]. The changes in zeta potential values might be related to the communal nanoparticle hinges [47,48].

### 4.4. Toxicity of Chitosan-Based Nanoparticles of Metarhizium anisopliae against Plutella xylostella

The dose–mortality response of *P. xylostella* larval instars to different concentrations of *M. anisopliae* mycelium–chitosan nanoparticles proved the toxicity of these nanoparticles against *P. xylostella.* The toxicity of nanoparticles is caused by different mechanisms such as the production of reactive oxygen species, oxidative stress, membrane disruption, protein unfolding and inflammation [49]. The higher doses of nanoparticles were toxic against all larval instars of *P. xylostella.* These results are consistent with the findings of Namasivayam et al. [14], who observed a similar dose–mortality relationship for *N. rileyi*–chitosan nanoparticles against different larval instars of *S. litura.* The LC_50_ values of nanoparticles showed an increasing trend with an increase in larval age. Such a trend in larval mortality might be related to the forbearance of larval instars as they grow. The median lethal concentration (LC_50_) of *M. anisopliae*–chitosan nanoparticles against second instar *P. xylostella* larvae under laboratory conditions was 14.44 ppm, which is different from the LC_50_ *B. brongniartii* Fe^0^NPs (58 ppm) observed against second instar *S. litura* larvae observed by Xu et al. [19]. Wang et al. [18] reported the median lethal concentration (LC_50_) value of 19.17 for *I. fumosorosea* Fe^0^NPs against second instar *B. tabaci* nymphs. Banu and Balasubramanian [29] found the LC_50_ value of 0.79 ppm for *B. bassiana* AgNPs against second instar larvae of *Aedes aegypti*. Santos et al. [50,51] also discussed and elaborated on the synthesis and toxicity of nanoparticles mediated by entomopathogenic fungi. The possible mechanism of action of *M. anisopliae*–chitosan nanoparticles can be explained by different phenomena or hypotheses, such as: (1) enhanced degradation of the insect cuticle or delayed ecdysis in response to *M. anisopliae*–chitosan due to a combined action of extracellular hydrolytic enzymes produced by fungi known for their effect on an insect cuticle [41,52,53,54]; or (2) decreased efficiency to convert the consumed food into a growth and energy source through the possible diversion of energy from growth to the detoxification process [10,19,20,21,22,23,24,25,26,27,28,29,30,31,32,33,34,35,36,37,38,39,40,41,42,43,44,45,46,47,48,49,50,51,52,53,54].

## 5. Conclusions

In summary, our findings provide a detailed explanation of the preparation and characterization of *M. anisopliae*–chitosan nanoparticles. The results also suggest that *M. anisopliae*–chitosan nanoparticles are bioactive against *P. xylostella* and can potentially be used within biorational *P. xylostella* management programs. However, further research is required to evaluate the effect of *M. anisopliae*–chitosan nanoparticles on other insects (pests as well as natural enemies) to determine the specificity and bio-safety of *M. anisopliae*–chitosan nanoparticles.

## Figures and Tables

**Table 1:** Details of different *M. anisopliae*–chitosan nanoparticles and *M. anisopliae* treatments used in the bio-activity studies.

Treatments	Treatment Description	Concentration
T1	*M. anisopliae*–chitosan nanoparticles	31.25 ppm
T2	*M. anisopliae*–chitosan nanoparticles	62.5 ppm
T3	*M. anisopliae*–chitosan nanoparticles	125 ppm
T4	*M. anisopliae*–chitosan nanoparticles	250 ppm
T5	*M. anisopliae*–chitosan nanoparticles	500 ppm
T6	*M. anisopliae* conidial suspension	10^6^ conidia/ml
T7	Chitosan nanoparticles	200 ppm
T8	Control (ddH_2_O)	0

## Data Availability

The raw data supporting the conclusion will be made available by the corresponding author on request.

## Supplementary Materials

The following are available online at https://www.mdpi.com/article/10.3390/microorganisms10010001/s1, Figure S1: The colony morphology and conidial morphology of different *Metarhizium anisopliae* isolate SM036, Figure S2: Concentration mortality response of 2nd instar *Plutella xylostella* larvae to *Metarhizium anisopliae* SM036, Figure S3: Concentration mortality response of 2nd instar *Plutella xylostella* larvae to *Metarhizium anisopliae*–chitosan nanoparticles, *Metarhizium anisopliae* conidia, and chitosan nanoparticles under laboratory conditions, Figure S4: Concentration mortality response of 2nd instar *Plutella xylostella* larvae to *Metarhizium anisopliae*–chitosan nanoparticles, *Metarhizium anisopliae* conidia, and chitosan nanoparticles under semi-field conditions.
