# Supplementary material for: Synthesis of Metarhizium anisopliae–Chitosan Nanoparticles and Their Pathogenicity against Plutella xylostella (Linnaeus)
Source: Microorganisms. 2021 Dec 21;10(1):1. doi: 10.3390/microorganisms10010001 (PMC8781626; doi:10.3390/microorganisms10010001)
Supplement: Supplementary file 1 [file microorganisms-10-00001-s001.zip › microorganisms-1492009-supplementary.pdf]

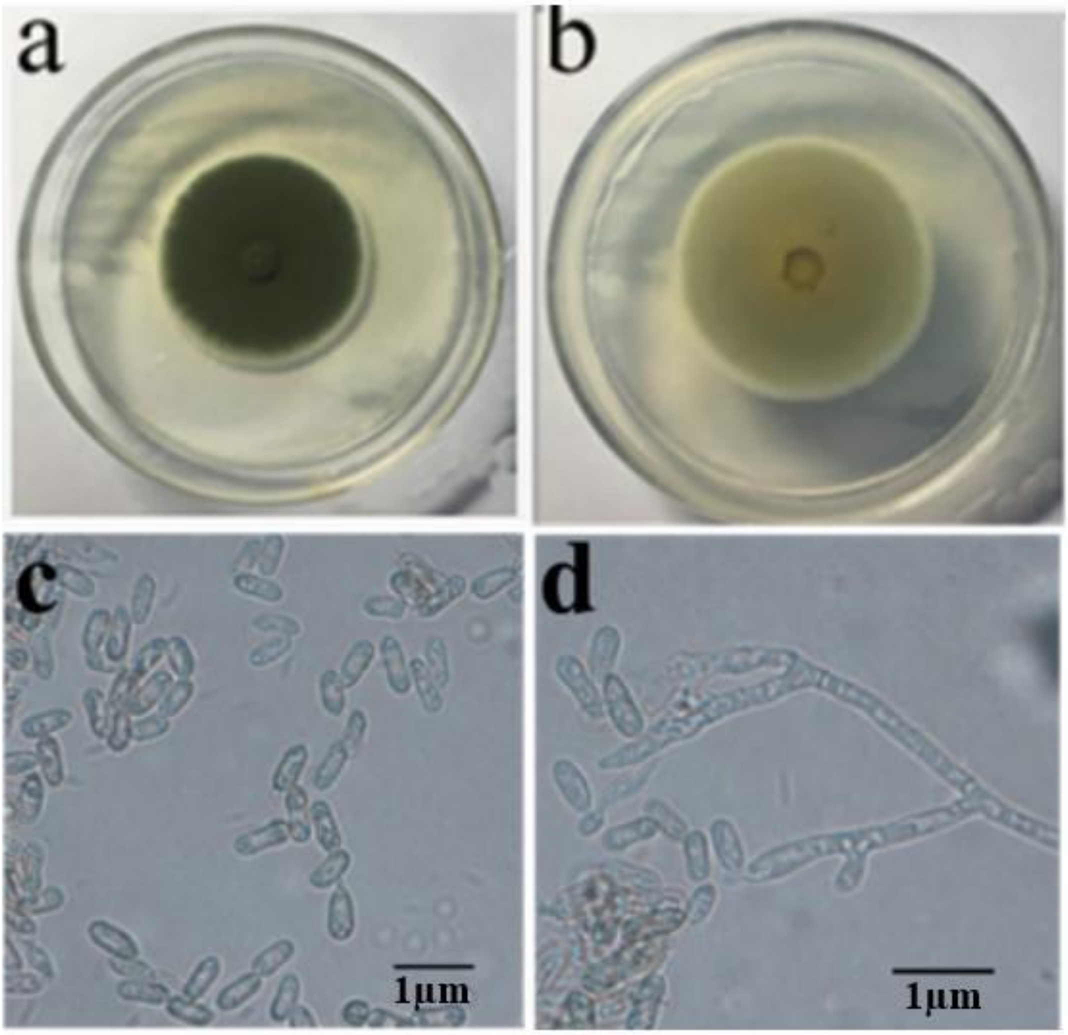

Supplementary Figure S1. The colony morphology and conidial morphology of different *Metarhizium anisopliae* isolate SM036. a, b: Colony morphology at 7 days old; c, d: Conidia of SM036.

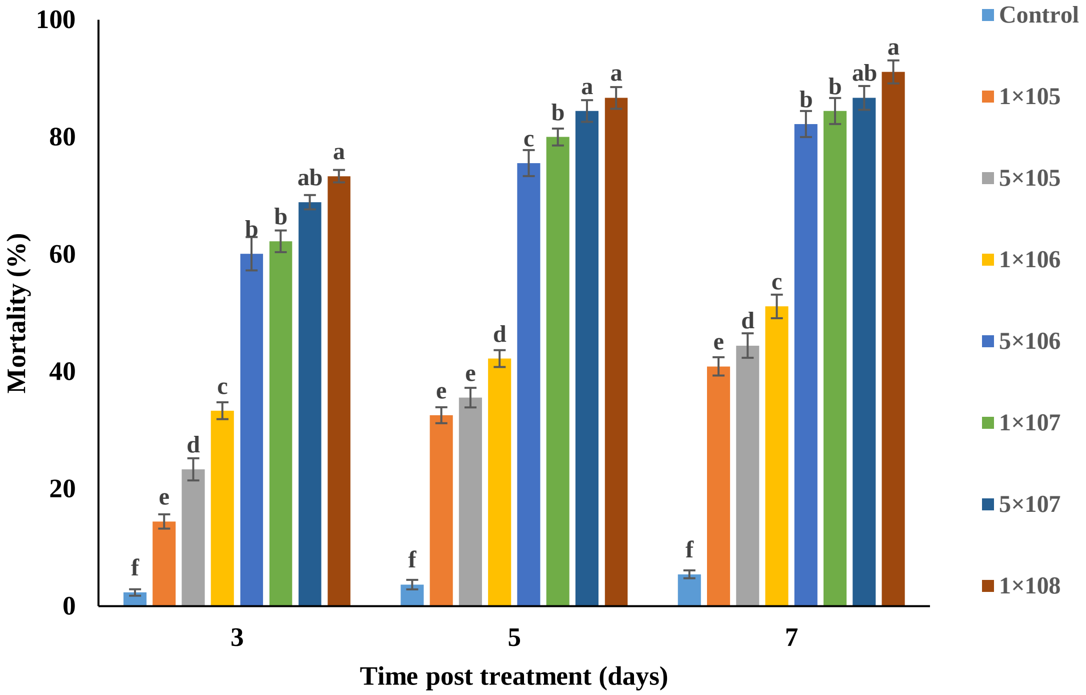

Supplementary Figure S2. Concentration mortality response of 2nd instar *Plutella xylostella* larvae to *Metarhizium anisopliae* SM036.

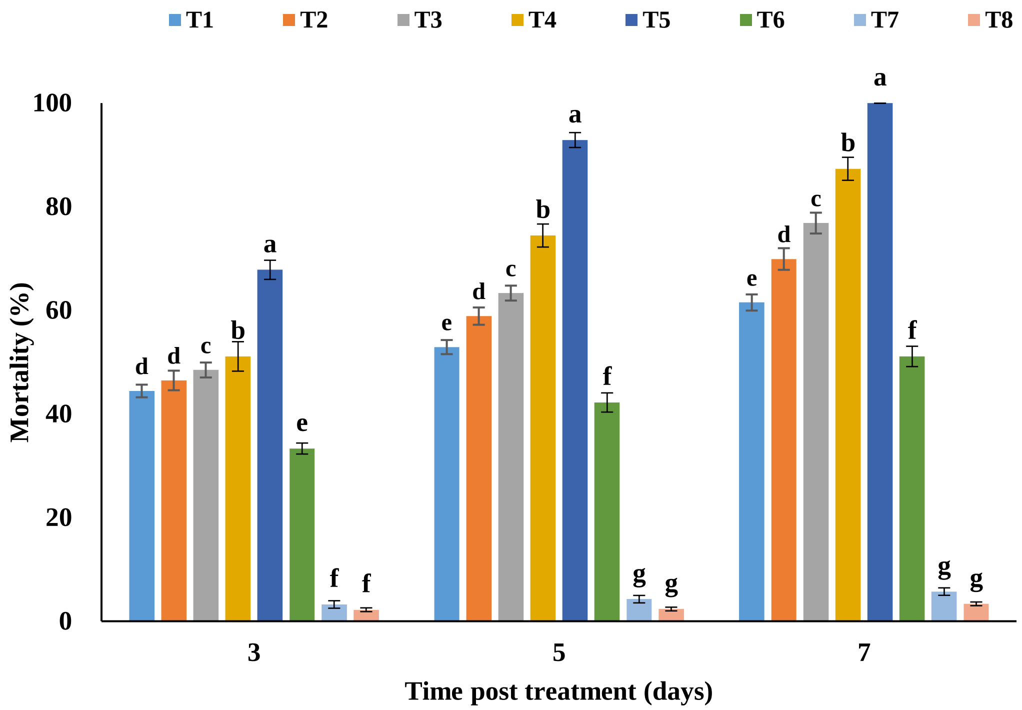

Supplementary Figure S3. Concentration mortality response of 2nd instar *Plutella xylostella* larvae to *Metarhizium anisopliae*-chitosan nanoparticles, *Metarhizium anisopliae* conidia; and chitosan nanoparticles under laboratory conditions. T1 : *M. anisopliae* chitosan nanoparticles 31.25 ppm; T2 : *M. anisopliae* chitosan nanoparticles 62.5 ppm; T3 : *M. anisopliae* chitosan nanoparticles 125 ppm; T4 : *M. anisopliae* chitosan nanoparticles 250 ppm; T5; *M. anisopliae* chitosan nanoparticles 500 ppm; T6 : *M. anisopliae* conidial suspension 1×10^6 conidia/ml; T7 : chitosan nanoparticles 200 ppm; and T8 : Control (ddH_2O).

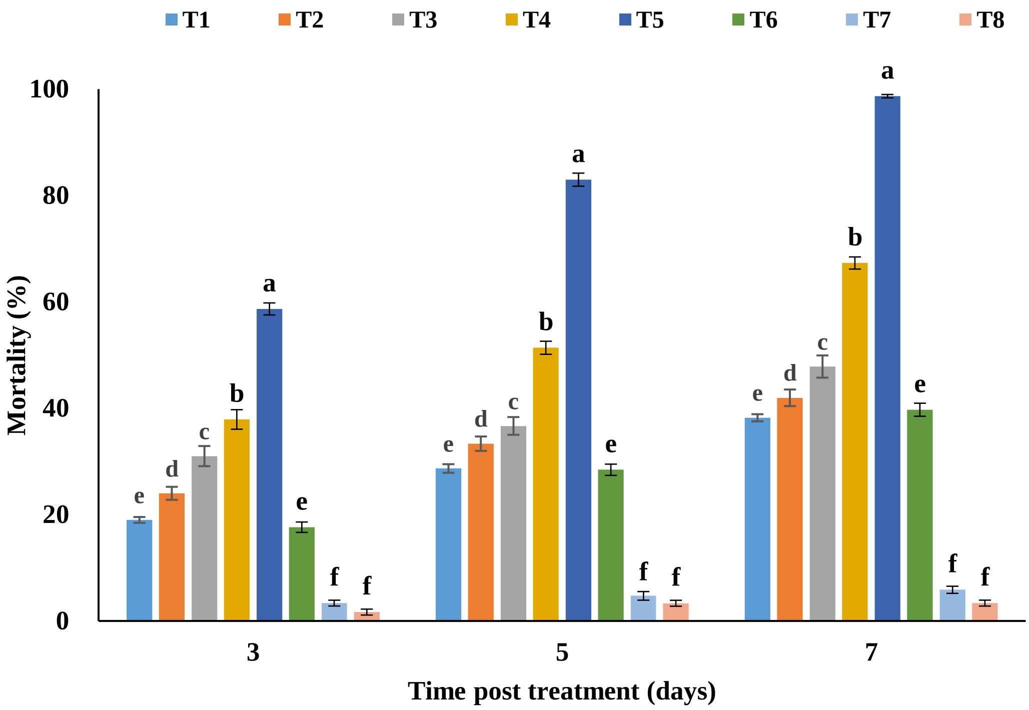

Supplementary Figure S4. Concentration mortality response of 2nd instar *Plutella xylostella* larvae to *Metarhizium anisopliae*-chitosan nanoparticles, *Metarhizium anisopliae* conidia; and chitosan nanoparticles under semi-field conditions. T1 : *M. anisopliae* chitosan nanoparticles 31.25 ppm; T2 : *M. anisopliae* chitosan nanoparticles 62.5 ppm; T3 : *M. anisopliae* chitosan nanoparticles 125 ppm; T4 : *M. anisopliae* chitosan nanoparticles 250 ppm; T5; *M. anisopliae* chitosan nanoparticles 500 ppm; T6 : *M. anisopliae* conidial suspension 1×10^6 conidia/ml; T7 : chitosan nanoparticles 200 ppm; and T8 : Control (ddH_2O).
